# Cystic Echinococcosis in Spain: Current Situation and Relevance for Other Endemic Areas in Europe

**DOI:** 10.1371/journal.pntd.0000893

**Published:** 2011-01-25

**Authors:** Francisco A. Rojo-Vazquez, Javier Pardo-Lledias, Marcelo Francos-Von Hunefeld, Miguel Cordero-Sanchez, Rufino Alamo-Sanz, Ana Hernandez-Gonzalez, Enrico Brunetti, Mar Siles-Lucas

**Affiliations:** 1 Universidad de León, Facultad de Veterinaria, Departamento de Sanidad Animal, Campus de Vegazana, León, Spain; 2 Hospital General de Segovia, Segovia, Spain; 3 Sevicio de Cirugía General y Digestiva, Hospital Nuestra Señora de Sonsoles, Avila, Spain; 4 Medicina Interna, Hospital Universitario de Salamanca, Salamanca, Spain; 5 Agencia de Proteccion de la Salud y Seguridad Alimentaria, Junta de Castilla y León, Valladolid, Spain; 6 Instituto de Recursos Naturales y Agrobiología, Consejo Superior de Investigaciones Científicas, Salamanca, Spain; 7 Division of Infectious and Tropical Diseases, University of Pavia, IRCCS S. Matteo Hospital Foundation, WHO Collaborating Centre for Clinical Management of Cystic Echinococcosis, Pavia, Italy; London School of Hygiene & Tropical Medicine, United Kingdom

## Abstract

Cystic echinococcosis (CE) remains an important health problem in many regions of the world, both where no control measures have been implemented, and where control programs have been incompletely successful with ensuing re-emergence of the disease. In Spain, official data on CE show an increase in the proportion of intermediate hosts with CE during the last few years, and autochthonous pediatric patients have been reported, a sign of active local transmission of disease. A similar picture emerges from data reported to the European Food Safety Authority by other European countries. Nevertheless, several crucial aspects related to CE that would help better understand and control the disease have not been tackled appropriately, in particular the emergence of infection in specific geographical areas. In this respect, while some data are missing, other data are conflicting because they come from different databases. We review the current situation of CE in Spain compared with areas in which similar problems in the CE field exist, and offer recommendations on how to overcome those limitations. Specifically, we believe that the introduction of national registries for CE with online data entry, following the example set by the European Registry for Alveolar Echinococcosis, would help streamline data collection on CE by eliminating the need for evaluating and integrating data from multiple regions, by avoiding duplication of data from patients who access several different health facilities over time, and by providing much needed clinical and epidemiological data that are currently accessible only to clinicians.

**Key Learning Points**

- Cystic echinococcosis (CE) remains one of the main zoonoses in both developing and developed countries, due to its complex clinical presentation, and causes a substantial number of cases in some areas.
- Recent analyses have shown that CE is a re-emerging disease with a remarkable economic impact in developed countries such as Spain.
- In spite of numerous studies, evidence-based and standardized/agreed approaches are still needed to define appropriate strategies for the epidemiological evaluation, immunodiagnosis, and clinical management of CE, among other aspects.
- The need for a continuous, homogeneous, and well-defined source of epidemiological data on human CE, improving the current EFSA reports, and modeling national registries after the European Registry for Alveolar Echinococcosis, is emphasized.

**Five Key Papers in the Field**

1. Brunetti E, Kern P, Vuitton DA; Writing Panel for the WHO-IWGE (2010) Expert consensus for the diagnosis and treatment of cystic and alveolar echinococcosis in humans. Acta Trop 114: 1–16.
2. Brunetti E, Junghanss T (2009) Update on cystic hydatid disease. Curr Opin Infect Dis 22: 497–502.
3. Budke CM, Deplazes P, Torgerson PR (2006) Global socioeconomic impact of cystic echinococcosis. Emerg Infect Dis 12: 296–303.
4. Kern P, Bardonnet K, Renner E, Auer H, Pawlowski Z, et al. (2003) European echinococcosis registry: human alveolar echinococcosis, Europe, 1982–2000. European Echinococcosis Registry. Emerg Infect Dis 9:343–349.
5. Craig PS, Budke CM, Schantz PM, Li T, Qiu JJ, et al. (2007) Human echinococcosis: a neglected disease? Trop Med Health 35: 283–292.

**Recommendations**

1. Notification of human CE and echinococcosis surveillance should be compulsory at the national level in European countries.
2. More detailed and uniform surveillance and confirmation criteria of cases for CE and echinococccosis in animals and human patients should be established in Europe.
3. Tools for the detection of CE applicable to epidemiological, diagnostic, and follow-up purposes should be standardized and widely agreed upon.
4. Genotyping of *Echinococcus granulosus* isolates from human patients and from wild hosts should be carried out for a proper assessment of the current epidemiological situation in Europe.

## Introduction

**Figure 1:**
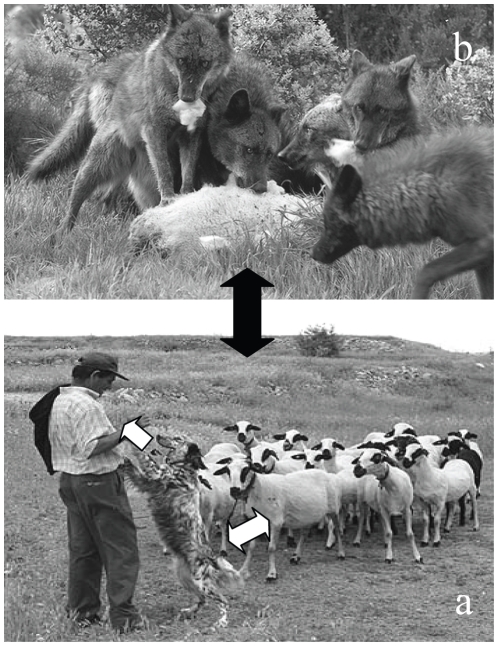
Epidemiology of *Echinococcus granulosus* in Spain. The main domestic cycle is maintained between dogs and sheep, with man as accidental intermediate host (a; white arrows). Wild cycles could occur between wolves, wild boars, and cervids, among others (b). The sylvatic cycle could be occasionally peridomestic (black arrow), since the G1 genotype, the most frequent in sheep and human patients, has been found in isolates from wild animals.

*Echinococcus granulosus* is a cestode whose larval stage causes cystic echinococcosis (CE) in livestock, wild animals, and humans. CE is acquired by ingesting eggs, originating from the faeces of definitive hosts (dogs, wolves, and other carnivores), that harbour the adult *E. granulosus* worms in their small intestine (Figure 1). CE is a neglected disease and the cause of significant losses in endemic areas [1], [2].

Where the importance of CE has been recognized, control programs have been implemented, leading to a drastic reduction in its prevalence. This has also caused the discontinuation of control measures and the exclusion of CE from the list of notifiable diseases.

Several publications have documented the re-emergence of CE in different European areas, e.g., Wales [3] and Spain [4], where high incidence rates of echinococcosis in dogs and new CE autochthonous cases in young people have been recently reported. As a result, CE has a renewed importance in Europe [5]. This has also been recognized by the European authorities through directive 2003/99/EC, in which CE is a disease to be reported to the European Food Safety Authority (EFSA).

This re-emergence should raise our awareness of crucial and incompletely elucidated aspects of this disease. Here, we present the latest data about CE in Spain and the suggestions from an expert panel about issues that have not been addressed, with a look at other countries that share the problem.

## Methods

We ran a Medline search using “cystic echinococcosis” and “hydatid” combined with “epidemiology”, “diagnosis”, and “control programs” as keywords. Additional articles were obtained from non-SCI journals published in Spain, Portugal, Italy, etc. Individual articles and other pieces of information were not excluded with regard to potential biases, since one of the goals was also to discuss said biases.

Statistics on CE in Spain and Europe were retrieved from the Ministerio de Sanidad y Consumo (http://www.msps.es), the Ministerio de Medio Ambiente y Medio Rural y Marino, Spain (http://www.mapa.es), and the EFSA (http://www.efsa.europa.eu). The review also drew on conference proceedings, original research conducted by the authors, and discussions in specific forums (e.g., the Conferences of the Spanish Hidatidology Association held in 2008 and 2010 at Salamanca, Spain).

## Cystic Echinococcosis in Animals: Epidemiological Changes

The lack of reliable statistics on the epidemiology of echinococcosis in dogs makes it difficult to compare prevalence rates before and after the application of control programs in Spain. A similar picture is found in other European countries, in which data about dog infection rates are scarce and difficult to interpret. Monitoring and notification of echinococcosis in dogs is not compulsory in Europe, and this information is not available to the EFSA.

The most complete data published to date about Spain are those from the CE control campaign in the northern Spanish region of La Rioja [6]. This campaign started in 1986, and CE prevalence in the definitive host, measured by necropsy of stray and unwanted dogs, was 7% at the beginning of the program, and 0.2% in 2000. A later study performed on shepherd dogs using *E. granulosus* coproantigen detection in the region of Alava, close to La Rioja, showed 8% of dogs to be positive [7]. Coproantigen ELISA tests to detect echinococcosis in definitive hosts are well established and documented [8], meaning that an active transmission of the parasite actually occurs in defined environments nowadays.

A similar situation following the discontinuation of respective control programs can be found in other European regions, such as Wales, where prevalence in rural dogs increased from 3.4% in 1989 to 8.1% in 2002 [3]. Although the information that can be gleaned from the above-mentioned studies is scarce, their findings suggest that rural and semi-rural dog populations are still at risk of infection with *E. granulosus* in these and in other regions of Europe where similar control campaigns had taken place.

A second point of concern is the role of wild definitive hosts in the epidemiology of *E. granulosus*. Infection with *E. granulosus* has been recently reported in the Iberian wolf [9], [10]. Although sample sizes are small, these authors claim that prevalence of echinococcosis in wolves, whose number increased in Europe in recent years due to protection policies (http://ec.europa.eu/environment/nature/conservation), might be higher than 14% in some regions of Spain [10]. More importantly, all positive wolves harbored gravid worms from the G1 strain [10], the cause of most human infections (reviewed in [11]). Wolves parasitized with *E. granulosus* have also been found in Belarus, Italy, Finland, and Bulgaria [12]–[15], with prevalence rates from 11.5% to 36%. Wild definitive hosts may contribute to the spread of human and animal CE in domestic cycles (Figure 1), and their role deserves further study in specific areas. Additional factors, such as the economic, social, and cultural conditions, sheep rising in extensive or semi-extensive (non-confined) systems, and transhumance may all facilitate the cross-talk of wild cycles with domestic environments.

Moreover, the prevalence of CE in domestic animals reported in recent studies on control campaigns differs from official data in some countries. In Spain, a CE prevalence of 0.98% for ovine and goats is officially recorded in 2000, while data recovered by personnel working in CE control programs showed 20 times that prevalence for sheep in specific Spanish regions, such as La Rioja, for the same year [6]. Official data show that from 2000 to 2008 (Table 1), bovine CE has decreased from 0.97% in 2000 to around 0.5% in 2008. Swine CE has been slowly decreasing from 2000 to 2008, although this data refers to intensively raised animals only. When data from extensively raised animals are available, prevalence rates are much higher compared to those in intensive (confined) farming animals. For example, in 2007 0.81% of extensively raised pigs had CE, compared with 0.02% of pigs maintained under intensive raising (Table 1). The higher prevalence found in extensive farm systems compared with intensively raised animals in pigs could also extend to other extensively raised CE hosts, e.g., sheep, which hosts the G1 human-infective strain. Sheep and goat CE remained below 2% from 2000 to 2007, but data from 2008 show that 3.68% of slaughtered animals were infected. Potential biases affecting data from 2008 and not affecting previous reports, e.g., the total number of inspected animals, are excluded, since the EFSA reports from 2007 and 2008 show that the number of inspected sheep and goats in Spain was higher in 2007 than in 2008. Similarly, other sources of error that might affect CE rates, such as the average animal's age, are excluded since no specific campaign for the slaughtering of old animals—with a higher likelihood of presenting visible cysts—had been carried out in Spain in that period. CE levels above 2% in sheep have also been reported to the EFSA in 2008 (http://www.efsa.europa.eu/en/scdocs/scdoc/1496.htm) in Bulgaria (4.3%), Italy (11.3%), Poland (6.7%), and Romania (5%). High levels of sheep CE were also reported in Portugal and Greece in 2007 (9.4% and 3.9%, respectively; http://www.efsa.europa.eu/en/scdocs/scdoc/223r.htm).

**Table 1:** Prevalence (%) of cystic echinococcosis in livestock and wild animals in Spain from 2000 to 2008.

Animals	Year								
	2000	2001	2002	2003	2004	2005	2006	2007	2008
Bovine	0.97	0.79	0.70	0.59	0.59	0.70	0.75	0.50	0.52
Swine (intensive)	0.20	0.04	0.03	0.03	0.07	0.03	0.05	0.02	0.03
Swine (extensive)	NA	NA	NA	NA	NA	NA	0.49	0.81	0.37
Sheep and goats	0.98	1.18	1.01	0.67	0.46	0.57	0.45	0.57	3.68
Wild boar	NA	NA	NA	NA	NA	0.04	0.06	0.13	0.17
Cervids	NA	NA	NA	NA	NA	NA	0.01	0.01	0.05

Source: Ministerio de Medio Ambiente y Medio Rural y Marino, Spain. NA, not available.

The potential role of wild intermediate hosts in the maintenance and spreading of CE into domestic environments is of interest to Spain. Data provided to the EFSA from Spain represent a proportion of randomly inspected hunted animals for human consumption. However, the detection of fertile cysts in wild boar suggest that this species may be involved in the epidemiology of *E. granulosus*, particularly considering that large amounts of carcass are available to dogs and wolves during the hunting season. The recent population increase of wild boar in Spain, and the DNA analysis showing that they harbor the G1 strain, indicates the importance of this wild host for public health in Spain [16] and in other European countries [17]. Estimated prevalence rates of CE in wild boars have only been reported in the last four national reports in Spain (from 2005 to 2008), showing an increase from 0.04% to 0.17% (Table 1). Cervid CE in Spain has been evaluated only from 2006, showing a low prevalence with a small increase in the last reported year (Table 1). Although the role of cervids in domestic-transmitted CE is probably minor due to the possible restriction to European cervids of the *E. granulosus* G10 genotype (reviewed in [18]), more detailed genotyping studies should be conducted to rule out G10 human infection or cervid infections with *E. granulosus* genotypes other than G10.

G7 may also play a role in human infections. It has been found in pigs, goats, and humans in Austria, Yugoslavia, Romania, Poland, Spain, and Turkey [19]–[21]. The importance of this and other genotypes in the epidemiology of CE is not well known, so molecular typing of human isolates of *E. granulosus* deserves to be systematically carried out in several areas of Europe.

## Current Situation of Human CE in Spain

In Spain, human CE has been a notifiable disease from 1982 to 1996. The Spanish official statistics showed a human CE incidence of 2.52 cases per 100,000 inhabitants in 1982. Control programs were started between 1986 and 1990 in different regions of the country, and were mainly based on the periodic treatment of dogs with praziquantel. This, and the strict control of carcasses after the occurrence of animal diseases such as spongiform encephalopathy in cattle and blue tongue in sheep, resulted in the decrease of human CE incidence to 1.01/100,000 inhabitants in 1996 (reviewed in [22]). From 1996 onwards, human CE cases registered at the national level have been those spontaneously declared by the different regions to the state authorities, and this may represent a problem, as stated by other authors from different European countries [5], [23]. In Spain, a comparison of notified cases with data from hospital records has shown that human CE case numbers have been clearly underestimated in the last 10 years (e.g., [4]). Thus, and for specific regions of Spain e.g., Castilla-León, the declared CE cases were 2.69/100,000, while those calculated from hospital records was four times higher for the same period [4]. Similarly, the latest surgical CE rates in Greece, Portugal, and Italy [23]–[26] were 12/100,000, 12.2/100,000, and up to 9.8/100,000 inhabitants per year, respectively, while notification of human CE to the EFSA from 2004 to 2008 from the same countries has been approximately ten times lower.

**Figure 2:**
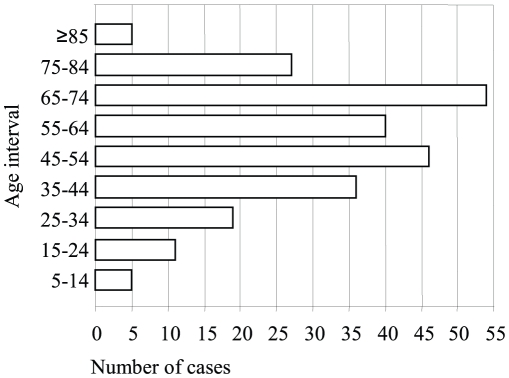
Age distribution of cystic echinococcosis human cases reported in Spain in 2006. Source: National Statistics Institute. Five autochthonous CE cases are found in the age range 5–14 years, indicating active transmission of the parasite. The rest of the cases are distributed as follows: 11 cases among 15- to 24-year-old patients, 19 among those aged 25–34, 36 among those aged 35–44, 46 among those aged 45–54, 40 among those aged 55–64, 54 among those aged 65–74, 27 among those aged 75–84, and five among patients aged 85 years old or more.

In addition, an increase of autochthonous cases of human CE was officially reported in 2006, with 0.54 cases per 100,000 inhabitants at the national level in Spain, this being the highest rate in the last 7 years (Table 2). More importantly, several pediatric cases were detected over that time period, indicating an active transmission of the parasite [27] (Figure 2). Re-emergence of human CE is also found in several countries where specific control programs have been applied in the past (reviewed in [1], [28]). This means that risk factors associated with human infection in different areas should be specifically assessed. In Spain, these risk factors were enumerated before the cessation of respective control campaigns by Campos-Bueno et al. [29]. Due to potential changes in the epidemiology of the parasite transmission, they should be re-evaluated in patients acquiring the infection in the last ten to 15 years in defined areas, as suggested by a study recently done in Germany [5]. Unfortunately, this re-evaluation cannot be performed with the currently available information from EFSA, since that does not include traceability of human cases related with crucial aspects such as occupational and other risks (e.g., hunting). The autochthonous character of human cases, an important factor for decisions on control measures, should in theory be assessed with the EFSA data, but their reliability is weakened by the fact that some countries, including Spain, have reported all cases from 2004 to 2008 as domestic. This is difficult to believe, since a sizeable proportion (30%–60%, depending on the hospital) of CE cases from Spanish hospital records correspond to immigrants from endemic countries (M. Siles-Lucas, J. Pardo-Lledias, and A. Hernandez-Gonzalez, unpublished data), as expected of a country where immigrants are more than 10% of the general population.

**Table 2:** Reported cases per 100,000 inhabitants of cystic echinococcosis in Spain from 2000 to 2008.

Source	Year								
	2000	2001	2002	2003	2004	2005	2006	2007	2008
EDOs	0.45	0.45	0.48	0.43	0.39	0.37	0.54	0.40	0.40
EFSA	NA	NA	NA	NA	<0.01	0.20	0.20	0.30	0.20
SIM	0.10	0.02	0.08	0.06	0.02	<0.01	0.00	0.06	0.03
CMBS	2.04	1.75	1.75	1.74	1.80	1.39	NA	NA	NA
Castilla-León	1.62	1.70	2.84	2.11	2.00	1.88	2.97	1.90	NA

Source: Ministerio de Sanidad, Spain, from the databases “Enfermedades de Declaración Obligatoria” (EDOs), “Sistema Microbiológico de Información” (SIM), and “Conjunto Mínimo Básico de Datos” (CMBS); European Food Security Authority (EFSA); and Consejeria de Sanidad y Bienestar Social de la Junta de Castilla y León (Castilla-León). NA, not available.

A comparison of CE human cases presented in three different Spanish databases, together with the cases notified to the EFSA and those notified by authorities in a specific Spanish region (Castilla-León) for the period 2000–2008, is shown in Table 2.

## Problems Peculiar to CE and Limitations of the EFSA Reports: Relevance for the European Endemic Zone

Short-term, periodic de-worming of dogs was one of the key points of control programs in Spain, but as it has been discontinued, deparasitation of dogs is now done once or twice a year, thus not covering the much shorter prepatent period of *E. granulosus* adult worms if present. This may result in the survival of the parasite in specific dog populations that actively shed the parasite. This seems to be the case in Spain, since animal CE has re-emerged and human CE also involves pediatric patients, and may well apply to other European countries with similar epidemiological scenarios.

Although the infection in dogs is probably the first indication of an active transmission of the parasite, data about infection rates in this and other *E. granulosus* definitive hosts are not notifiable to the EFSA. Interestingly, however, some data about fox infection with *Echinococcus multilocularis* are detailed in the EFSA annual report.

Data about dog prevalences are only found in a few publications, and are frequently biased since there is no consensus on what should be measured (e.g., dog subpopulation, seasonality, age structure, number of samples, etc.) or on the diagnostic method to use. In most studies, these variables are not or are only partly considered; thus, interpretation of tendencies or of specific time-point data is impossible. In any case, the results of studies on prevalence in dogs from several European countries are worrisome. Those done in Spain, and more specifically in Alava on shepherd dogs using coproantigen detection [7], and data from Wales [3] and some Italian regions (reviewed in [30]), show dogs to be infected at a rate of about 10%.

Data from farm animal CE prevalence in Spain reported to the EFSA are obtained on all slaughtered animals by inspection each year. Even so, these are probably underestimations, as the vast majority of slaughtered animals—especially sheep—are less than 1 year old, less than the time needed for the oncosphere to develop into a visible lesion; only 4% of slaughtered sheep in 2008 in Spain were older than 12 months (http://www.mapa.es/es/ganaderia). An example of this underestimation can be found in the official data from the Spanish Castilla-León region, where reports on sheep CE was 3.87% for animals under 12 months of age, and 38.47% for sheep older than 5 years (http://www.salud.jcyl.es/sanidad/cm?locale=es_ES&textOnly=false). Here, the problem is related to the animal's age at the time of sacrifice, although data are probably showing the true tendency of CE in Spain, since all slaughtered animals are reported. Nevertheless, underestimations have consequences on policy making, resulting in the minimization of the true importance of the problem and in playing down the need of new and better documented studies.

In other European countries, CE data on farm animals reported to EFSA are limited to small numbers far inferior to the total of slaughtered animals in the same period. For example, Italy reports data of CE in sheep for 358,602 animals in 2008, while FAOSTAT data shows that in 2008 6,189,767 sheep were slaughtered in Italy (http://faostat.fao.org/site/603/default.aspx#ancor). It is impossible to know whether the reported animals were randomly selected from several areas or are from a specific area inside the country in which CE incidence is high, medium, or low, and this makes discerning trends, if any, impossible.

Important additional information to assess CE epidemiology such as age, geographical origin, or raising practices for animals is not collected. In our opinion, these data would be easily obtained in countries where an animal tracking system has been imposed by the authorities, like in Spain (http://www.mapa.es/es/ganaderia/pags/rega/riiaremo.htm).

Data from CE in wild animals are reported to EFSA by some European countries, representing, in the case of Spain, only a proportion of randomly inspected hunted animals. We are not aware of the mandatory character of inspections or of the proportion of animals inspected in other European countries. Unfortunately, the extent and significance of infection of wildlife for humans cannot be assessed with the currently available data. A clear protocol on how to collect information about CE rates in wild animals, as well as typing of *E. granulosus* isolates, should be outlined.

Regarding human CE data, Spain has three different databases: the “Enfermedades de Declaración Obligatoria” or EDOs (declarable diseases), the “Sistema de Información Microbiológica” (SIM, system of microbiological information), and the “Conjunto Mínimo Básico de Datos” (CMBD, minimum database). All are maintained by the Spanish Ministry of Health, but each database gives a different number of human CE cases for the same year (Table 2). The CMDB database shows the highest CE rates, although it only reports CE patients who underwent surgery. Nevertheless, a variable amount of cysts remain clinically silent or are not surgically removed [31].

Furthermore, the EDOs data should be transferred to the EFSA, but Spanish data of human CE in the EFSA reports also differ from EDOs, SIM, and CMBD (Table 2). Differences between EDOs and EFSA-declared data might be due to the method used for case confirmation, which should be histopathology. Thus, EFSA figures may represent only a fraction of EDOs. Nevertheless, discrepancies between both databases are also related with relative proportion of patients along time, since they do not indicate the same trend over time.

Notification systems in other European countries may suffer from the same problems. Additionally, in some European countries such as Italy, the notification for human CE has also ceased to be mandatory. The ensuing underreporting leads authorities to think that CE is not an important health problem, which in turn makes measurement of disease burden even more difficult.

Human CE data in the EFSA reports are sometimes accompanied by tables with age distribution of cases, showing the occurrence of pediatric cases, but these are not assigned to a specific country. Even if they were, a second problem would remain, the autochthonous character of human cases, which has epidemiological and policy making implications. Autochtonous cases are reported for some countries in the EFSA bulletin, but as mentioned above, Spain has declared all cases from 2004 to 2008 as domestic. These data are hardly credible for the reasons mentioned above (M. Siles-Lucas, J. Pardo-Lledias, and A. Hernandez-Gonzalez, unpublished).

To make matters worse, CE has many clinical peculiarities with epidemiological implications. The understanding of the natural history of liver cysts has greatly improved with the use of ultrasound and of a standardized classification [32]–[34], but these important details are not collected in the notification form. Further, given the chronic nature of their illness and the frequent relapses, patients with CE often move from one treatment center to another, which also contributes to unreliable statistics. In any case, traceability of human cases, as well as other aspects important for the re-evaluation of the epidemiological situation of CE in Europe, such as occupation and previous or current residence in an endemic area, should be obtained.

## Conclusions

Available information on CE is incomplete and is insufficient to assess properly its epidemiology in Spain and other European countries. CE importance tends to be underestimated due to underreporting and to the lack of compulsory notification. In spite of this, Benner et al. [35] attempted to calculate the overall economic losses due to human and animal CE in Spain in 2005. Assuming no underreporting, they estimated an annual loss of €148,964,534. This shows the need for increased monitoring and control of CE in those countries where CE continues to affect certain areas despite several control initiatives. We believe that these limitations could be overcome, at a relatively low cost, with the establishment of national registries for CE with Internet electronic data entry, following the example set by the European Registry for Alveolar Echinococcosis [36]. The registry would help streamline data collection on CE by eliminating the need for evaluating and integrating separate data from multiple regions, by avoiding duplication of data from patients who access several different health facilities over time, and by providing much needed clinical and epidemiological data that are currently accessible only to single clinicians. The registry could be used as a tool to prioritize control measures for what is essentially a preventable disease. Unsurprisingly, these suggestions are similar to those offered by the Scientific Panel on Animal Health and Welfare in Europe to the EFSA regarding *E. multilocularis* [37]. Indeed, the problems outlined in this review regard not only CE, but other neglected zoonotic diseases as well, such as cysticercosis.

CE warrants more attention from clinicians, but their coordination with veterinarians and policy makers is also required to implement a more effective approach to CE control.
